# Assessment of barriers to optimal cancer control in adult cancer treatment centres in Cameroon

**DOI:** 10.3332/ecancer.2023.1601

**Published:** 2023-09-21

**Authors:** Berthe Sabine Esson Mapoko, Andreas Frambo, Yauba Saidu, Esther Dina Bell Mbassi, Etienne Atenguena, Kareen Azemafac, Emily Kobayashi, Lionel Tabola, Glenda Nkeng, Anne Sango, Anne Marthe Maison, Sidonie Ananga Noa, Ambroise Ntama, Ruth Rosine Meka’h Mapenya, Rachel Tayou, Francine Kouya, Glenn Mbah, Pelagie Douanla, Celestin Fonkwa, Martin Essomba Biwole, Zacharie Sando, Albert Mouelle Sone, Paul Ndom

**Affiliations:** 1 Faculty of Medicine and Biomedical Sciences, University of Yaoundé I, Yaoundé 99322, Cameroon; 2 Clinton Health Access Initiative, Yaoundé 99322, Cameroon; 3 Institute for Global Health, University of Siena, Siena 53100, Italy; 4 Faculty of Medicine and Pharmaceutical Sciences, University of Douala, Douala 99322, Cameroon; 5 Faculty of Health Sciences, University of Buea, Buea 99322, Cameroon; 6 Faculty of Health Sciences, University of Dschang, Dschang 99322, Cameroon; 7 Mbingo Baptist Hospital, Bamenda 99322, Cameroon; 8 Faculty of Health Sciences, University of Bamenda, Bamenda 99322, Cameroon; 9 Laquintinie Hospital oF Douala, Douala 99322, Cameroon

**Keywords:** assessment, barriers, cancer control, Cameroon

## Abstract

Approximately 20,745 new cases of cancer were registered annually with 13,199 (64%) deaths in 2020 in Cameroon. Despite the increasing cancer burden, there is a paucity of reliable data that can enhance decision-making for cancer control in Cameroon. This assessment was, therefore, designed to generate data that may enable stakeholders, policymakers and funders to make data-driven decisions on cancer control. We conducted a cross-sectional survey in July 2020, which enabled us to collect data on key cancer variables from six adult cancer treatment centres in Cameroon. The key components of the assessment included case detection, service availability, human resource capacity, cost of chemotherapy and radiotherapy, the safety of chemotherapy sessions, data systems, patient education, palliative care, funding for chemotherapy and chemotherapy stock. Data were compiled and analysed using Microsoft Excel 2016. Data from four of the 6 sites show that 1,636 new cases were recorded representing an annual case detection rate of 11.8%. All the six assessed facilities offered chemotherapy services, 5/6 (83.3%) offered surgery for cancers, while just 1 (16.7%) offered radiotherapy services. In addition, none offered nuclear medicine services for cancer care and treatment. Similarly, none of the facilities had the WHO-recommended number of human resources for optimal cancer care. Overall, there were only 6 medical oncologists, 2 surgical oncologists, 3 radiation oncologists and 14 oncology nurses providing services across the 6 cancer treatment centres. Treatment services are expensive for an average national, with a complete course of chemotherapy followed by radiotherapy costing ~XAF 1,240,000 (~$2,480). None of the survey facilities had a recommended safe biosafety cabinet and clean room for the preparation of chemotherapies, rendering the preparation of chemotherapies suboptimal and hazardous. Data collection tools were manual, relatively available and very different across all the surveyed sites and the interval for data collection and transmission was collectively undefined. Optimal cancer care in adult cancer treatment centres is limited by several health systems and socio-economic factors. The identification of these barriers has enabled the formulation of action-oriented interventions, leveraging on the recently adopted national strategy for the prevention and control of cancers in the country.

## Introduction

Cameroon, like most resource-challenged countries, is experiencing a significant increase in cancer-related morbidity and mortality. According to estimates from the World Health Organisation, International Agency on Research in Cancer, over 20,745 new cases of cancer and approximately 13,199 (67.95%) cancer-related deaths occurred in 2020 [1]. The burden appears to be particularly high in women. For instance, in 2020, nearly 60% of 20,745 new cases were recorded in women [1]. In both sexes, the top five cancers are breast, cervix uteri, prostate, non-Hodgkin lymphoma and Kaposi sarcoma [1].

In response to this cancer situation, the Government of the Republic of Cameroon, through its Ministry of Public Health, was created in 1990 and later re-organised in 2002, a National Committee for the Fight Against Cancer (NACFAC) [2]. In 2019, NACFAC developed a 5-year national strategic plan to reduce cancer-related morbidity and mortality by 2024, by 10%, and attenuating its socio-economic impact on the development of the country. Progress towards attaining this goal has been slow, because of several factors including limited access to affordable cancer medication, scarcity of cancer specialists, dilapidated infrastructure, outdated platforms, high treatment discontinuation rates and underfunding of interventions amongst others [2]. To date, limited efforts have been invested to explore the barriers to holistic and optimal cancer care in the country. This study was, therefore, designed to investigate the key obstacles to accessing optimal cancer care in the functional oncology services in Cameroon. Specifically, we sort to describe cancer care and treatment capacity across six approved treatment sites; evaluate human resource capacity involved in the management of cancers; identify potential factors affecting access to cancer care and treatment with a focus on chemotherapy, radiotherapy and surgery; evaluate existing patient follow-up systems, including patient education and palliative care; describe existing cancer data collection and reporting systems and to understand the funding landscape for chemotherapy.

## Methods

We conducted a cross-sectional study in July 2020 in the six adult cancer treatment centres of Cameroon. These were Yaoundé General Hospital (YGH), Yaoundé Central Hospital (YCH), Douala General Hospital (DGH), Douala Laquintinie Hospital (DLH), Bonassama District Hospital (BDH) in Douala and Mbingo Baptist Hospital (MBH) in Bamenda.

In collaboration with key stakeholders, we designed, pre-tested and validated a structured questionnaire (see Supplementary Data) that captured data on various aspects of cancer treatment in the study sites. The questionnaire had seven sections. The first section captured health facility information, including the address, the type of facility, the category of the facility and the bed capacity amongst others. The second section captured information on the number of health staff available as well as the proportion of staff that have been capacitated on oncology. The third and fourth sections covered information relating to accessing cancer care and treatment with a focus on chemotherapy, surgery, radiotherapy and nuclear medicine. The fifth section essentially focused on the follow-up of cancer patients including preparation and administration of chemotherapy sessions, patient education, tracking patients on chemotherapy and palliative care amongst others. The sixth section captured information on data systems for cancers and finally, the seventh section focused on stock management for chemotherapy.

The tool was reviewed and approved during a joint meeting, which brought together various stakeholders, including the United Nations International Children's Emergency Fund, the International Atomic Energy Agency (IAEA), the NACFAC and the Clinton Health Access Initiative (CHAI). Following this approval, the study team under the leadership of the cancer programme, proceeded to collect data from six adult cancer treatment centres in Cameroon. The data were collected by visiting the sites, interviewing a focal point of the staff and consulting registers. We then entered the collected data into a pre-established database and subsequently analysed it using Microsoft Excel. Table 1 presents the characteristics of the responders in the different centres.

## Results

Overall, we surveyed one private faith-based hospital and five state-owned hospitals. In these centres, we sought to assess the availability and provision of various cancer care and treatment services, including chemotherapy, radiotherapy, nuclear medicine, surgery and palliative care.

### Cancer service availability

Table 1 shows the cancer care services that were provided at the study sites during the time of the study. As can be seen, all six surveyed facilities offered adult chemotherapy services and palliative care. Surgery was offered by five of the six hospitals, while radiotherapy was offered by just one hospital. No nuclear medicine service is available (Table 2).

In adults, 1,636 new cases were diagnosed annually across four treatment sites, representing about 11.8% (1,636/13,830) of adult cancer case detection.

### Human resource capacity

Six medical oncologists, 2 surgical oncologists, 3 radiation oncologists and 14 oncology nurses were the trained human resource providing services across the 6 cancer treatment centres (Table 2). About 85% (143/168) of staff working in cancer treatment centres have not received prior training in cancer care (Table 3).

### Access to cancer treatment

Chemotherapy services require about XAF 67,000 (~$134) for pre-treatment investigations prior to starting chemotherapy. The average cost of one cycle of chemotherapy is XAF 165,500 (~$331) and six cycles of chemotherapy is averagely required for a complete curative cancer treatment. A full course of radiotherapy costs XAF 180,000 (~$316). This figure reduces when treatment is palliative. Complete chemotherapy followed by radiotherapy will cost ~XAF 1,240,000 (~$2,480) as illustrated in Table 4. In addition to this cost, which is paid out of pocket, patients will also cover transport, accommodation and meals amongst others.

### Safety of chemotherapy sessions

Chemotherapy sessions are essentially prepared by nurses, and pharmacy technicians when available. These staff have received on-the-job training for chemotherapy preparation and administration and they basically work on average 2–5 days per week and then change shifts. Personal protective equipment (PPE) for chemotherapy sessions seems to be lacking as only three hospitals (43%) had all the essential PPEs in use. The spaces allocated for the preparation of chemotherapy sessions are inadequate.

### Chemotherapy stock

All the six surveyed hospitals had a pharmacy where they could stock anticancer medicines. However, only four of the six surveyed sites had dedicated personnel who oversee chemotherapy stock. Overall, the sources of chemotherapy stock were pharmaceutical companies (43%), the National Drug Procurement Agency (CENAME) (29%) and private pharmacies (14%). The MBH procures through the CBCHS Central Pharmacy from abroad and other local private wholesalers. Only three of the six surveyed hospitals provided information on the current chemotherapy stock. Amongst them, two were entirely stocked out. Only two of the assessed hospitals had visibility on the quantity of procured versus used stock for 2019. Overall, the quantities procured were lesser than the quantities used. With stockouts in these hospitals, patients procure from private pharmacies where prices are significantly higher.

### Patient education

Disclosure of cancer status to patients is essentially done by the already overburdened oncologists and a few other medical specialists for adults. Flyers, posters and health talks are the main education and sensitisation materials. Patient follow-up is mainly with hospital registers and sometimes patient records.

### Palliative care

Palliative care services are available. Morphine (rarely available), dexamethasone, metoclopramide and diazepam are the main palliative medicines given to patients.

### Data systems

Data collection tools were manual, relatively available, and very different across all the surveyed sites. The interval for data collection was collectively undefined. Data reporting was infrequent as three sites (50%, (3/6)) reported data only when necessary.

### Funding for chemotherapy

Funding for chemotherapy procurement was provided by the state (22%), hospital-generated funds (33%) and non-governmental organisation like Max Foundation, Bayonne Hospital and Chemotherapy Solidarity (44%). None of the surveyed hospitals provided verifiable data on the amount spent on chemotherapy procurement. In one hospital, an anecdotal estimate of XAF 4,900,000 (~USD 8,604) was used to purchase anticancer medicines for a year. In the other hospitals, the data were either non-existent or unknown.

## Discussion

The purpose of this study was to provide a detailed outline of the *status quo* of cancer care and treatment in the six approved adult cancer care and treatment centres in Cameroon. The goal was to provide stakeholders, policymakers and funders with information that can enable them to understand the main factors affecting access to cancer care and treatment as this may galvanise the design and implementation of evidence-based interventions. Overall, we found that optimal cancer care and treatment in Cameroon is marred with several limitations, including inadequate adult chemotherapy, radiation therapy and nuclear medicine services. These findings are in line with those reported by Vanderpuye *et al* [3] and Stefan [4], who observed that resources allocated to cancer care and treatment services are inadequate, a challenge that will only continue to increase premature deaths from cancers [5]. Our findings suggest that urgent actions are needed to improve access to cancer care and treatment services in Cameroon. In the short to medium term, this need should focus on redressing access challenges to chemotherapy and radiotherapy in the six hospitals while in the long term, focus should be directed towards expanding chemotherapy and radiotherapy services to other regions of the country.

We also found that cancer care and treatment centres lacked the necessary human resource capacity for optimal cancer treatment and survival. For instance, only 6 medical oncologists, 3 radiation oncologists, 2 surgical oncologists and 14 oncology nurses were available across all 6 treatment centres in the country. The IAEA recommends training radiation/clinical oncologists who can prescribe both radiation and chemotherapy for common solid cancers and that the number of specialists needed should be based on the number of cancer patients [6]. The agency also recommends that to ensure proper coverage, each treatment centre should have at least two surgical oncologists, two radiation/clinical oncologists, two haematologist oncologists, etc. [6]. This was not the case in all the surveyed hospitals. Worst still, nearly 85% of staff working in the surveyed cancer treatment centres have not received prior training on cancer care. These limitations highlight the need for strengthening health resource capacity for cancer control through targeted interventions to immediately strengthen the skills of the existing health resource workforce in the short to medium term. Setting up a mechanism to increase the existing pool of oncologists (doctors and nurses) will be needed to curb the human resource challenges in the long run. As a result, deliberate investment by governments would be critical to ensure the availability of a trained and competent workforce to redress premature deaths resulting from cancer [5].

We also found that the cost of chemotherapy is largely beyond the financial capacity of the majority of the population, including 37.5% of the ones who are already living below the poverty threshold of XAF 931 (~$1.8) per day [7]. Indeed, our data show that a patient will have to spend an average of XAF 67,000 (~$134) for pre-treatment investigations, XAF 990,000 ($1980) for six-cycle chemotherapy course. In addition, where indicated, a patient will need to spend XAF 180,000 (~$316) for a complete radiotherapy session. Indeed, nationals that are living below the poverty line will need to save all on their monthly income of approximately XAF 28,309 (~$49) for over six months to be able to raise the amount needed for radiotherapy alone [7]. Given this financial challenge, the majority of people suffering from cancer requiring a curative chemotherapy followed by radiotherapy are likely to abandon treatment due to cost [8]. As such, rendering chemotherapy (pre-treatment investigations, hospital stay and medicines) and radiotherapy affordable may significantly improve access, thereby impacting survival rates after cancer treatment [9]. This may go a long way to prevent patients from abandoning treatment [5] or driving their families into dire poverty [10, 11]. Indeed, cost is one of the major reasons why many patients abandon their treatments in Africa as patients frequently must pay out of pocket to access care, incurring expenses that can be financially high [3, 5, 10, 11].

Patient education remains one of the critical components for achieving better survival rates with cancer treatment [12]. There is absolute need for building the capacity of healthcare workers, community workers and most importantly, the caregivers, on key elements for improving survival including disclosure of cancer status [13, 14]. Furthermore, conducting awareness and sensitisation interventions will indirectly improve patient education and facilitate adherence to cancer treatment [15].

Palliative care services are available for cancer and other diseases. However, very few drugs are being used for this purpose and these aren’t readily available. A substantial number of cancer patients are in need of palliative care seeing the late stage at which most cancers are being diagnosed in Cameroon. Studies have shown that over 80% of people in urgent need of palliative care live in low- and middle-income countries where basic health care is less well developed [16]. Palliative care service is generally not limited to what we found in this study such as the administration of palliative drugs. Hence, there are proposals on increasing the palliative health care package in the country [17–20].

Data systems for capturing and reporting information relating to cancer care and treatment are unstructured and embryonic. Indeed, there is a need for an overhaul of the existing data-capturing system to embrace harmonised and user-friendly tools including basic registers and monthly reporting forms for the short and medium term. Setting up a cancer registry at regional levels will facilitate the availability and synthesis of key indicators to enable decision-making in the long term [21]. The International Agency for Research on Cancer insisted on the fact that the unique value of cancer registry data is manifested in its contribution to decision-making in cancer control [22]. However, few countries in Africa have adequate cancer registries in place [22]. It was therefore recommended that funding new cancer registries and improving the quality of existing ones should be considered a high priority on the agenda of African health policy-makers, Cameroon inclusive [23]. Patient tracking mechanisms were weak suggesting the need to institute robust patient tracking mechanisms to monitor treatment progress and measure dropout rates [15].

Access to adequate financial resources appears to be the major barrier to the implementation of optimal cancer control interventions. Indeed, most surveyed facilities lack ready funds to secure resources for basic cancer services including chemotherapy. Consequently, it is not uncommon for these facilities to entirely rely on stakeholders to support the availability of chemotherapy medicines [24].

## Conclusion

The findings of the survey have enabled the identification of gaps in cancer control. The above findings suggest that to sustainably improve access to cancer care and treatment, a wide range of interventions targeting the major arms of cancer care and treatment will be required. The Government of Cameroon has demonstrated good political will in this line and has translated the recommendations into actionable interventions for implementation. This report complements the 5-year national strategic plan for prevention and cancer response and enhances the endeavour of the Ministry of Health.

## Conflicts of interest

AF, YS and EK work at CHAI which funds the current study. EDBM works at Laboratoire ROCHE as part of its ACCESS CANCER programme in Cameroon. However, Laboratoire ROCHE is neither closely nor remotely involved in the conception, writing or financing of this research work. The other authors do not have any conflicts of interest.

## Funding

This study was financed by the UBS Foundation through the CHAI.

## Author contributions

Conception and design: YS, AF, EK, BSEM, and NP.

Data collect: BSEM.

Data analysis and interpretation: AF, BSEM, and KA.

Manuscript writing: BSEM, AF, KA, TL, and GN.

Manuscript revision: BSEM, AF, YS, EK, EDBM, KA, TL, EA, AJFS, AMMM, SAN, RT, RRMM, AN, PD, FK, GM, CF, MEB, AMS, ZS, and PN.

All the authors approved the final version of the manuscript.

## Figures and Tables

**Table 1.** Characteristics of the respondents.

Variables	Site	Sex	Age	Title	Position	Years of service
Respondent 1	General Hospital of Douala	M	66	Radiotherapist	Head of Radiotherapy and Oncology Department	28
Respondent 2	General Hospital of Yaoundé	M	45	Medical oncologist	Head of Oncology Department	13
Respondent 3	Central Hospital of Yaoundé	F	39	Medical oncologist	Head of Oncology Department	2
Respondent 4	Douala Laquintinie Hospital	M	50	Oncology surgeon	Head of Oncology Department	17
Respondent 5	District Hospital of Bonassama	F	40	Medical oncologist	Head of Oncology Department	3
Respondent 6	Mbingo Baptist Hospital	F	52	Internist/medical oncology	Internal Medicine / Oncology Medical Supervisor	6

**Table 2.** Cancer service availability.

Variable	YGH	YCH	DGH	DLH	BDH	MBH
Year opening oncology unit	1997	2019	1997	2000	2017	2007
Oncology unit bed capacity	35	07	19	18	13	20
Palliative care	√	√	√	√	√	√
Adult chemotherapy	√	√	√	√	√	√
Adult surgery	√	√	√	√	X	√
Radiotherapy	X	X	√	X	X	X
Nuclear medicine	X	X	X	X	X	X

**Table 3.** Distribution of human resources for cancer care and treatment.

Variable	Number of personnel
Medical oncologists	06
Radiation oncologists	03
Surgical oncologists	02
Oncology nurses	14
Radio-physicists	02
Radio-technicians	09
Gynaecologists	61
General practitioners	05
Nurses	45
Assistant nurses	19
Secretaries	02
Total	168

**Table 4.** Average cost of complete curative cancer treatment.

SN	Service	Average cost/XAF	Frequency	Total/XAF
1.	Pre-treatment investigations	67,000	1	67,000
2.	Chemotherapy session	165,500	6	990,000
3.	Radiotherapy session	180,000	1	180,000
	Total			1,237,000

## Supplementary data

**Assessment of the barriers to optimal cancer care and treatment in Cameroon**

**Questionnaire Date July 2020**

**Table:** Section 1: Health facility information

1.1	Address	
	Name of health facility	
	Department/service:	
	Address (quarter/street):	
	Postal code:	
	City:	
	Region:	
	Website:	
	Tél 1:	
	Tél 2:	
	Fax:	
	E-mail:	
	Date of creation:	
1.2	Type	
	Public	
	Private	
	Confessional	
1.3	Health facility category	
	First category (Reference Hospital)	
	Second category (Central Hospitals)	
	Third category (Regional Hospitals)	
	Fourth category (District, CMA, CSI)	
1.4	Cancer care and treatment service capacity	
	Paediatric chemotherapy	
	Adult chemotherapy	
	Paediatric palliative care	
	Adult palliative care	
	Surgery for paediatric cancers	
	Surgery for adult cancers	
	Radiotherapy	
1.5	Total number of beds in the hospital	
	Number	
1.6	Number of beds reserved for cancer cases	
	Number	
1.7	Percentage of beds occupied by cancer patients?	
	Percentage	
1.8	Do you often reach full bed capacity?	
	Yes	
	No	
1.9	If yes, where do you hospitalise when in full capacity?	
	Different unit in hospital – specify	
	Another health facility – specify	

**Table:** Section 2: Human resource

2.1	Number of healthcare workers trained on cancer management	
	Medical oncologist	
	Paediatric oncologist	
	Radiotherapist	
	Gynaecologist/surgeon	
	GP medical doctor	
	Radiophysicist	
	Radio-technician	
	Nurse	
	Nurse-assistant	
	Psychosocial agent	
	Other – specify	
2.2	Number of healthcare workers involved in cancer care and treatment	
	GP medical doctor	
	Nurse	
	Nurse-assistant	
	Psychosocial agent	
	Other – specify	
2.3	Number of healthcare workers that have attended a workshop on diagnosis on management of cancers	
	GP medical doctor	
	Nurse	
	Nurse-assistant	
	Psychosocial agent	
	Other – specify	
2.4	**Date of the most recent seminar attended by health care worker?**	
	**Date**	
2.5	The most recent seminar attended concerned which cancer?	
	NA	
	**Paediatric cancers**	
	Burkitts	
	Wilms	
	Retinoblastoma	
	Rhabdomyosarcoma	
	ALL	
	AML	
	Hodgkins	
	Non Hodgkins	
	Neuroblastoma	
	Ewing’s sarcoma	
	Osteosarcoma	
	Other – specify	
	**Adult cancers**	
	Cervical	
	Breast	
	Prostate	
	Colorectal	
	Liver	
	Ovarian	
	Stomach	
	Pancreas	
	Uterus	
	Lung	
	NHL	
	Bladder	
	Skin	
	Other – specify	

**Table:** Section 3: Chemotherapy

Section 3A: Paediatric chemotherapy				Section 3B: Adult chemotherapy		
3a1	**Number of paediatric cancer cases diagnosed in 2019?**			3b1	**Number of adult cancer cases diagnosed in 2019?**	
	Burkitts				Cervical	
	Wilms				Breast	
	Retinoblastoma				Prostate	
	Rhabdomyosarcoma				Colorectal	
	ALL				Liver	
	AML				Ovarian	
	Hodgkins				Stomach	
	Non Hodgkins				Pancreas	
	Neuroblastoma				Uterus	
	Ewing’s sarcoma				Lung	
	Osteosarcoma				NHL	
					Bladder	
					Skin	
3a2	Number of cases that received chemotherapy in 2019?			3b2	Number of cases that received chemotherapy in 2019?	
	Burkitts				Cervical	Cervical
	Wilms				Breast	Breast
	Retinoblastoma				Prostate	Prostate
	Rhabdomyosarcoma				Colorectal	Colorectal
	ALL				Liver	Liver
	AML				Ovarian	Ovarian
	Hodgkins				Stomach	Stomach
	Non Hodgkins				Pancreas	Pancreas
	Neuroblastoma				Uterus	Uterus
	Ewing’s sarcoma				Lung	Lung
	Osteosarcoma				NHL	NHL
					Bladder	Bladder
					Skin	Skin
3a3	Number of children with cancers that completed chemotherapy treatment in 2019?			3b3	Number of adults with cancers that completed chemotherapy treatment in 2019?	
	Burkitts				Cervical	
	Wilms				Breast	
	Retinoblastoma				Prostate	
	Rhabdomyosarcoma				Colorectal	
	ALL				Liver	
	AML				Ovarian	
	Hodgkins				Stomach	
	Non Hodgkins				Pancreas	
	Neuroblastoma				Uterus	
	Ewing’s sarcoma				Lung	
	Osteosarcoma				NHL	
					Bladder	
					Skin	
3a4	**Number of treatment cycles per paediatric cancer type**			3b4	**Number of treatment cycles per cancer type**	
	Burkitts				Cervical	
	Wilms				Breast	
	Retinoblastoma				Prostate	
	Rhabdomyosarcoma				Colorectal	
	ALL				Liver	
	AML				Ovarian	
	Hodgkins				Stomach	
	Non Hodgkins				Pancreas	
	Neuroblastoma				Uterus	
	Ewing’s sarcoma				Lung	
	Osteosarcoma				NHL	
	Burkitts				Bladder	
					Skin	
					Cervical	
3a5	**Treatment protocols per paediatric cancer type**			3b5	**Treatment protocols per adult cancer type**	
	Burkitt's lymphoma				Prostate	
						
						
						
						
	Wilms tumour (nephroblastoma)				Breast	
						
						
						
						
	Retinoblastoma				Cervical	
						
						
						
						
	Rhabdosarcoma				Colorectal	
						
						
						
						
	ALL				Liver	
						
						
						
						
	AML				Ovarian	
						
						
						
						
	Hodgkin's lymphoma				Stomach	
						
						
						
						
	NHL				Pancreas	
						
						
						
						
	Neuroblastoma				Uterus	
						
						
						
						
	Ewing's sarcoma				Lung	
						
						
						
						
	Osteosarcoma				NHL	
						
						
						
						
					Bladder	
						
						
						
						
					Skin	
						
						
						
						
3a6	Average cost of pre-treatment work ups for paediatric cancers?			3b6	Average cost of pre-treatment work ups for adult cancers?	
	CBC				CBC	
	SGPT				SGPT	
	SGOT				SGOT	
	CREAT				CREAT	
	Glycaemia				Glycaemia	
	Electrolytes				Electrolytes	
	X-ray				X-ray	
	Ultrasound				Ultrasound	
	Others – specify				Others – specify	
3a7	Average cost of daily services for chemotherapy session in children?			3b7	Average cost of daily services for chemotherapy session in adults?	
	Nursing care				Nursing care	
	Hospitalisation				Hospitalisation	
	Adjuvant treatment				Adjuvant treatment	
	Others – specify				Others – specify	
3a8	**Average cost of chemotherapy session in children?**			3b8	**Average cost of chemotherapy session in adults?**	
	Burkitts				Cervical	
	Wilms				Breast	
	Retinoblastoma				Prostate	
	Rhabdomyosarcoma				Colorectal	
	ALL				Liver	
	AML				Ovarian	
	Hodgkins				Stomach	
	Non Hodgkins				Pancreas	
	Neuroblastoma				Uterus	
	Ewing’s sarcoma				Lung	
	Osteosarcoma				NHL	
					Bladder	
					Skin	
3a9	Do you organise multidisciplinary meetings for treatment initiation and follow-up?			3b9	Do you organise multidisciplinary meetings for treatment initiation and follow-up?	
	Yes				Yes	
	No				No	
3a10	If yes, how often?			3b10	If yes, how often?	
	Weekly				Weekly	
	Monthly				Monthly	
	Other – specify				Other – specify	
3a11	Who are the principal participants of these meetings?			3b11	Who are the principal participants of these meetings?	
						
						
						

**Table:** Section 4: Radiotherapy and nuclear medicine

**Section quarter A: Radiotherapy services**	
4a1	Do you carry out radiotherapy for cancer treatment ?
	Yes
	No
4a2	If no, where do you refer clients for radiotherapy?
	1.
	2.
	3.
4a3	Why is radiotherapy not offered?
	No staff
	No space
	Faulty equipment
	Absence of equipment
	Other
4a4	If yes, what is the cost of one session?
	Normal session cost
	Intensive session cost
4a5	How many sessions do you typically run in one week?
	Monday
	Tuesday
	Wednesday
	Thursday
	Friday
	Saturday
	Sunday
	Total
4a6	Do you carry-out radiotherapy sessions on weekends?
	Yes
	No
4a7	If No, when are they available during the week?
	NA
	Monday
	Tuesday
	Wednesday
	Thursday
	Friday
4a8	How many staff do you have in radiotherapy service?
	Radiotherapist
	GP medical doctor
	Radiophysicist
	Technical support staff
	Others
4a9	How many staff have been trained on radiotherapy?
	Radiotherapist
	GP medical doctor
	Radiophysicist
	Technical support staff
	Others
4a10	What is the name/mark on the devise currently in use?
	
	
4a11	What is the radioactivity source? Quelle est le type de source radioactive utilisée?
	
	
4a12	What is its half-life?
	
	
4a13	Do you often use this source beyond the stipulated time?
	Yes
	No
4a14	If yes, what is the maximum time ever used beyond the stipulated time?
	
4a15	How long has the current device been functioning?
	NA
	<5 years
	>5 years
	>10 years
	Other – specify
4a16	Where do you procure ‘radioactive source’?
	NA
	Name of local supplier
	Name of foreign supplier
	Others – specify
4a17	Do you have sufficient stock for the next semester?
	NA
	Yes
	No
4a18	When was the last radioactive source exhausted?
	NA
	<1 year
	>1 year
	Specify
4a19	Where do you refer clients during periods of exhausted stock?
	1.
	2.
	3.
4a20	What is the number of clients who were eligible for radiotherapy in 2019?
	
4a21	What is the number of patients that effectively received radiotherapy treatment in 2019?
	
4a22	Do you have equipment for quality control?
	Yes
	No
4a23	How often do you conduct quality control sessions?
	
4a24	Do you have a maintenance plan?
	Yes
	No
4a25	How often do you carry-out maintenance of current equipment?
	
4a26	Is there a Nuclear medicine unit?
	Yes
	No
4a27	Do you have a nuclear medicine specialist?
	Yes
	No
4a28	If no, are there plans for a nuclear medicine unit?
	Yes
	No
4a29	If yes, what is the estimated time for the unit to be operational?
	<2 years
	2–5 years
	>5 years

**Table:** Section 4B: Surgery for cancers

4b1	Do you carry out surgery for cancer treatment?
	Yes
	No
4b2	If no, where do you refer clients for surgery for cancer treatment?
	1.
	2.
	3.
4b3	Why is surgery not offered?
	No staff
	No space
	Faulty materials
	Absence of equipment
	Other
4b4	Average cost of surgical intervention?
	Normal surgery
	Complex surgery
4b5	How many surgeries do you typically carry-out in a week?
	Monday
	Tuesday
	Wednesday
	Thursday
	Friday
	Saturday
	Sunday
	Total
4b6	Do you conduct cancer surgeries on weekends?
	Yes
	No
4b7	If no, when do you conduct them within the week?
	NA
	Monday
	Tuesday
	Wednesday
	Thursday
	Friday
4b8	How many staff work at the surgical department for the management of cancers?
	Surgeon – please specify type and number
	Gynaecologists – please specify type and number
	Surgical nurses
	Nursing assistants
	Others – please specify
4b9	Number of staff trained on cancer surgery?
	Surgeon – please specify type and number
	Gynaecologists – please specify type and number
	Surgical nurses
	Nursing assistants
	Others – please specify
4b10	Can you evaluate the success of your surgeries in the management of cancers?
	Complications following surgery
	Death rates after surgery
	Others – please specify
4b11	Can you suggest any elements to improve upon the surgical management of cancers in your institution?
	
	

**Table:** Section 5: Patient follow-up and palliative care

5.1	Preparation and administration of chemotherapy	
5.1.1	Number of personnel involved in chemotherapy session preparation	
	Pharmacist	
	Nurse	
	Nurse assistant	
	Other – please specify	
5.1.2	Number of personnel trained on the preparation of a chemotherapy session	
	Pharmacist	
	Nurse	
	Nurse assistant	
	Other – please specify	
5.1.3	Frequency of rotation of personnel involved in the preparation of chemotherapy sessions	
	1 day	
	2 days	
	3 days	
	4 days	
	5 days	
	Other – please specify	
5.1.4	Personal protective material available for session preparation	
	Gloves	
	Gowns	
	Goggles	
	Visières	
	Face masks	
	Laminar flow cabinet	
	Others – please specify	
5.1.5	Space of preparation of chemotherapy	
	Bed side	
	Special room	
	Common room	
	Laminar flow cabinet	
**5.2**	**Patient education**	
5.2.1	Who does the disclosure of cancer status to the caregivers – informs the parent of the child/accompanying the adult patient about his or her cancer status (e.g., diagnosis, treatment, side effects and their management)?	
	Oncologist	
	Other specialist – specify	
	Residents	
	GP medical doctor	
	Nurse	
	Psychosocial worker	
	Nurse aid	
	Other – specify	
5.2.2	Who does disclosure of cancer status to the child/adult patient – informs the child/adult of his/her disease (e.g., diagnosis, treatment, side effects and their management)?	
	Parents	
	Oncologist	
	Other specialist – specify	
	Residents	
	GP medical doctor	
	Nurse	
	Psychosocial worker	
	Nurse aid	
	Other – specify	
5.2.3	How many of these ‘educators’ are available in the health facility?	
	Oncologist	
	Residents	
	GP medical doctor	
	Nurse	
	Psychosocial worker	
	Nurse aid	
	Other – specify	
5.2.4	Have these ‘educators’ been trained on disclosure of cancer status? Yes/No	
	Oncologist	
	Residents	
	GP medical doctor	
	Nurse	
	Psychosocial worker	
	Nurse aid	
	Other – specify	
5.2.5	If so, how many educators have received training?	
	Oncologist	
	Residents	
	GP medical doctor	
	Nurse	
	Psychosocial worker	
	Nurse aid	
	Other – specify	
5.2.6	Is there any material used to educate parents/caregivers about cancer and its care?	
	Yes	
	No	
5.2.7	If so, indicate how they are being used (e.g., documents, counselling sessions, text messages and video messages) and when they are published	
	**Type of material**	**Method of use**
1		
2		
3		
5.2.8	Is there any material used to educate people with cancer?	
	Yes	
	No	
5.2.9	If yes, indicate how they are being used (e.g., documents, counselling sessions, text messages and video messages) and when they were released	
	**Type of material**	**Method of use**
1		
2		
3		
5.2.10	Do you organise cancer education fora, with parents/families of people with cancer	
	Yes	
	No	
5.2.11	If yes, how often do you organise them?	
	Every month	
	Every quarter	
	Every semester	
	Every year	
	Others – specify	
5.2.12	On average, how many parents attend such a forum?	
	Average number	
5.2.13	Do you organise cancer education fora, with people living with cancer	
	Yes	
	No	
5.2.14	If yes, how often do you organise them?	
	Every month	
	Every quarter	
	Every semester	
	Every year	
	Other – specify	
5.2.15	Are there other forums to share information about childhood/adult cancers with community members	
	Yes	
	No	
5.2.16	If yes, indicate which ones?	
	Schools	
	Religious institutions	
	Media	
	Associations	
	Others – specify	
5.2.17	On average, how many times a year do you organise these sessions to educate the community?	
	Number	
**5.3**	**Follow-up of patients on chemotherapy**	
5.3.1	Do you conduct is the tracking of patients who do not show up for treatment?	
	Yes	
	No	
5.3.2	If yes, how do you estimate the dropout rate? (source, numerator and denominator)?	
	NA	
	Source	
	Numerator	
	Denominator	
5.3.3	What was the number of patients who discontinued treatment in 2019?	
	Men	
	Women	
	Children	
5.3.4	What was the dropout rate in 2019?	
		
5.3.5	Number of cancer patients who do not show up for more than 4 weeks from the scheduled date for treatment. NOTE: Please do not answer this question if you are aware of the loss to follow-up in 2019	
	Men	
	Women	
	Children	
5.3.6	Number of cancer patients who missed an appointment, were contacted and returned to hospital in 2019	
	Men	
	Women	
	Children	
**5.4**	**Palliative care**	
5.4.1	Do you carry-out palliative care?	
	Yes	
	No	
5.4.1.1	How many people are involved in palliative care?	
	Number	
5.4.1.2	Who are the specialists involved in palliative care in your hospital?	
		
5.4.2	What are the palliative care services offered?	
	Male cancers	
	Female cancers	
	Paediatric cancers	
	Congestive heart failure	
	HIV/AIDS	
	Liver diseases	
	Renal disorders	
	Sickle cell	
	Dementia	
	Other – specify	
5.4.3	What are the essential medicines which you regularly use for palliative care	
	NA	
	Atropine	
	Benztropine	
	Clonazepam	
	Dexaméthasone	
	Halopéridol	
	Hyoscine	
	Métoclopramide	
	Morphine	
	Naloxone	
	Promethazine	
	Diazepam	
	Midazolam	
	Propofol	
	Autres	
5.4.4	If you do not offer palliative care, where do you refer clients for palliative care?	
	1	
	2	
	3	
5.4.5	Why is palliative care not offered?	
	Lack of trained personnel	
	Lack of infrastructure	
	Absence of essential medicines	
	Other – specify	

**Table:** Section 6: Data systems

6.1	What tools do you use for data collection?
	Cancer registry
	Monthly report form
	Service register
	Other – specify
6.2	How often do you prepare reports/statistics?
	Daily
	Weekly
	Monthly
	Every semester
	Annual
	When necessary
	Other – specify
6.3	How often do you transmit your reports to a superior level?
	Daily
	Weekly
	Monthly
	Every semester
	Annual
	When necessary
	Other – specify
6.4	Do you organise morbid and morality review meetings? i.e., meetings to with staff to examine the performance and other issues
	Yes
	No
6.5	If yes, kindly indicate the frequency?
	Daily
	Weekly
	Monthly
	Every semester
	Annual

**Table:** Section 7: Chemotherapy stock management

7.1	Is there a pharmacy dedicated for anticancer medicines?	
	Yes	
	No	
7.2	What is the number of persons in charge of chemotherapy stock management?	
	Number	
7.3	What is the number of persons trained on the chemotherapy stock management?	
	Number	
7.4	Where does the hospital procure anticancer medicines?	
	CENAME	
	Private pharmacy	
	Pharmaceutical company – specify	
	Other – specify	
7.5	What is the source of funding for chemotherapy medicines?	
	State	
	Hospital funds	
	Stakeholders – specify	
	Others – specify	
7.6	Averagely, how much does the hospital spend each year on cancer medication?	
	2017	
	2018	
	2019	
7.7	Do all patients get drugs from the hospital?	
	Yes	
	No	
7.8	If no, kindly indicate the reasons-	
	
	
	
7.9	Where else do patients source for cancer medications?	
	Private pharmacy	
	From another hospital	
	Importation	
	Other – specify	

**Table:**

Name of medicine	Dosage (mg)	Form	Sales unit	Sale price	Current stock	Qty procured in 2019	Qty used in 2019	Number of days in the month when the stock was zero											
								**2019**											
**Paediatric medicines**								**J**	**F**	**M**	**A**	**M**	**J**	**J**	**A**	**S**	**O**	**N**	**D**
Asparaginase	10^3^U	Vial	1																
Cytarabine	100	Vial	1																
Cyclophosphamide	1,000	Vial	1																
Doxorubicin	50	Vial	1																
Etoposide	50	Capsules	20																
Mercaptopurine	50	Tablet	25																
Methotrexate	25	Tablet	100																
Vincristine	1	Vial	1																
Daunorubicine	20 or 50	Vial	1																
Ifosfamide	2,000	Vial	1																
Actinomycine D	0.5	Vial	1																
Bléomycine	15	Vial	1																
Filgrastim	0.3 or 0.48	SPR	1																
																			
																			
																			
																			
																			
																			
																			
																			
																			

**Table:**

Name of Medicine	Dosage (mg)	Form	Sales unit	Sale price	Current stock	Qty procured in 2019	Qty used in 2019	Number of days in the month when the stock was zero											
								**2019**											
**Adult medicines**								**J**	**F**	**M**	**A**	**M**	**J**	**J**	**A**	**S**	**O**	**N**	**D**
Anastrazole	1	Tablets																	
Bicalutamide	50	Tablets																	
Bleomycine	15	Vial																	
Capecitabine	500	Tablets																	
Carboplatine	450	Vial																	
Cisplatine	50	Vial																	
Cytarabine	100	Vial																	
Docetaxel	80	Vial																	
Doxorubucine	50	Vial																	
Epirubicin	50	Vial																	
Fluorouracil	500	Vial																	
Gemcitabine	1,000	Vial																	
Leucovorin (folic acid)	15	Tablets																	
Methotrexate	50	Vial																	
Oxaliplatine	100	Vial																	
Paclitaxel	100	Vial																	
Vinblastine	10	Vial																	
Tamoxifene	20	Tablets																	
Letrozole	2.5	Tablets																	
Cyclophosphamide	500 or 1,000	Vial																	
Irinotecan	40 or 100	Vial																	
Sorafenib	200	Tablets																	
Filgrastim	0.3 or 0.48	SPR																	
Autre																			
																			

**Table:** Contacts

Focal contact name	Position	Profession	Tel	E-mail
				
				
				
				
				
				
				
				

**Signatures**

**Name and date-assessor Name and date: Health facility lead**
